# Numerical Simulation and Theoretical Analysis of Flexural Strengthening of Undamaged RC Beams with Steel Strand Mesh-Reinforced ECC

**DOI:** 10.3390/ma19132854

**Published:** 2026-07-03

**Authors:** Danju Song, Xiaoxiao Zhou, Yong Liang, Mingchen Wang, Hanyu Shi, Jiao Song, Ke Li

**Affiliations:** 1 Department of Architecture and Art Design, Henan Geology Mineral College, Zhengzhou 451464, China; 13903860361@163.com; 2 Department of Civil Engineering, Zhengzhou University, Zhengzhou 450001, China; zxx15890503448@163.com (X.Z.); 15039691038@163.com (Y.L.); 3 Wellington Institute, Zhengzhou University, Zhengzhou 450052, China; shy060605@163.com (H.S.); zdsongj@163.com (J.S.)

**Keywords:** reinforced concrete (RC), steel strand mesh-reinforced ECC, finite element (FE), flexural strengthening

## Abstract

The effects of practical parameters on the flexural behavior of reinforced concrete (RC) beams strengthened with steel strand mesh-reinforced engineered cementitious composite (ECC) were investigated, based on the finite element (FE) simulation. First, an FE model for strengthened RC beams was developed. The model was validated by comparing it with existing experimental data. Subsequently, the model was employed for parametric analysis on the flexural performance of the strengthened beams. The results showed that steel strand mesh-reinforced ECC significantly enhanced the flexural capacity, stiffness, and ductility of the RC beams, with improvements ranging from 7.81% to 61.84%, 6.35% to 40.90%, and 5.92% to 50.16%, respectively. As the reinforcement ratio of longitudinal steel strand, ECC thickness, and cracking strength increased, the flexural capacity increased. However, an increase in the reinforcement ratio of the longitudinal steel bars and the section height of the RC beam reduced the improvement in flexural capacity. The increase in the thickness of the strengthening layer and reinforcement ratio of the longitudinal steel strand enhanced the improvement of stiffness. Differently, an increase in the reinforcement ratio of longitudinal steel strand, concrete strength, and height of the RC beam diminished the improvement of stiffness. The enhancement of ductility increased with the concrete strength. Finally, formulas for calculating the bearing capacity and stiffness of RC beams strengthened with steel strand mesh-reinforced ECC and the limit of steel strand quantity were proposed. These formulas agreed well with experimental and numerical simulation FE results.

## 1. Introduction

Currently, many RC members have exhibited varying degrees of damage due to long-term loading, overload usage, environmental corrosion, and natural disasters [1,2]. This rendered them unable to meet safety and serviceability requirements. To avoid the enormous costs and environmental impacts caused by demolishing and rebuilding existing structures, employing appropriate strengthening measures is necessary. However, the existing strengthening methods still have some limitations [3]. The interface enlargement strengthening method has issues such as a high workload for wet operations, a long curing period, and significant occupation of construction space. In the concrete replacement strengthening method, problems include prolonged wet operation time and unsuitability for structures with relatively low concrete grades. Achieving more efficient enhancement of mechanical performance for RC structures fundamentally relies on advanced strengthening materials. Consequently, scholars worldwide have conducted research on material-based strengthening methods for RC members [4,5].

Currently, the strengthening methods of steel strand mesh-polymer mortars have gained widespread application [6,7]. This is because, compared with resin-based strengthening systems, cementitious matrix-based systems have better compatibility with concrete substrates and are less sensitive to temperature variation because of the inorganic matrix and the thermal stability of steel reinforcement [8]. However, mortars are prone to cracking and exhibit poor ductility, often causing stress concentration in the steel strands and limiting their strength. Therefore, steel strand mesh must be used in combination with a substrate possessing superior ductility and crack-controlled capacity to develop its high-strength performance. ECC exhibits exceptional ductility, toughness, and crack-controlled capacities [9,10]. Research has demonstrated that RC beams strengthened with ECC or bar-reinforced ECC effectively enhanced flexural capacity, ductility, and crack resistance [11,12]. Therefore, the combination of steel strand mesh and ECC was proposed, named “steel strand mesh-reinforced ECC” [13,14].

Tests have been conducted on this composite material. Wang et al. [15] performed bending tests on steel strand mesh-reinforced ECC panels. The test results indicated that the panels exhibited excellent crack-controlled and deformation capacities. Increasing the reinforcement ratio of steel strands enhanced the flexural strength of the panels but reduced ductility. Wei et al. [16] investigated the seismic performance of RC columns strengthened with steel strand mesh-reinforced ECC. The test results exhibited a significant improvement in the seismic performance of RC columns after being strengthened. The steel strand mesh-reinforced ECC jacket demonstrated superior effectiveness compared to the ECC jacket in strengthening. Li et al. [17] conducted experimental studies on the flexural performance of non-destructive RC beams strengthened with steel strand mesh-reinforced ECC. The test results showed that the cracking load, bearing capacity, stiffness, and ductility of RC beams were improved significantly.

However, the impact of various factors on the mechanical performance of RC members strengthened with steel strand mesh-reinforced ECC has not been comprehensively explored. In recent years, nonlinear FE analysis has gained significant attention [18,19,20]. Due to limitations in experimental facilities and testing environments, traditional experimental methods can only address a portion of engineering problems. In contrast, numerical simulation methods can overcome these limitations. The accuracy and reliability of numerical simulation can be validated by comparing predicted results with test data.

To sum up, numerical simulation of RC beams strengthened with steel strand mesh-reinforced ECC was employed by ABAQUS Version 2022 software in this study. The aim was to fully investigate the influence patterns and underlying mechanisms of parameter changes on the strengthening effect of the steel strand mesh-reinforced ECC. The parameters included the ECC thickness, reinforcement ratio of longitudinal steel strands, cracking strength of ECC, ultimate tensile strength of ECC, longitudinal reinforcement ratio of the RC beam, concrete strength, and height of the RC beam. Additionally, simplified calculation methods for the bearing capacity and stiffness of strengthened RC beams were also proposed.

## 2. Finite Element Simulation

### 2.1. Material Constitutive Laws

Concrete and ECC were modeled using the built-in plastic damage model of ABAQUS [21,22]. The stress–plastic strain relationship for concrete was derived from the constitutive model specified in the Chinese Code [23]. The bilinear elastic–plastic model was used to express the stress–strain relationship for steel bars. The strands used in this study were fabricated from high-strength stainless steel. No prestressing force was applied, and the strands acted as passive reinforcement in the ECC. The relationship between the stress (*σ*_sw_) and the strain (*ε*_sw_) of steel strands employed the three-stage tensile constitutive relationship [14]:

(1) $$\sigma_{\mathrm{sw}}=\left\{\begin{array}{ll} E_{\mathrm{sw}1}\varepsilon_{sw} & 0\leq \varepsilon_{\mathrm{sw}}\leq \varepsilon_{\mathrm{sw}1}\\ \sigma_{\mathrm{sw}1}+E_{\mathrm{sw}2}\left(\varepsilon_{\mathrm{sw}}-\varepsilon_{\mathrm{sw}1}\right) & \varepsilon_{\mathrm{sw}1}<\varepsilon_{\mathrm{sw}}\leq \varepsilon_{\mathrm{sw}2}\\ \sigma_{\mathrm{sw}2}+E_{\mathrm{sw}3}(\varepsilon_{\mathrm{sw}}-\varepsilon_{\mathrm{sw}2}) & \varepsilon_{\mathrm{sw}2}<\varepsilon_{\mathrm{sw}}\leq \varepsilon_{\mathrm{swu}} \end{array}\right.$$

where *E*_sw1_, *E*_sw2_, and *E*_sw3_ are the elastic modulus of the steel strand at three stages, respectively; ε_sw1_, ε_sw2_, and *ε*_swu_ are the strain values of 40%, 60%, and 100% of the ultimate tensile strain of steel strands, respectively; *σ*_sw1_, *σ*_sw2_, and *σ*_swu_ are the corresponding stress values corresponding to ε_sw1_, *ε*_sw2_, and *ε*_swu_, respectively.

It should be noted that the modulus reported in Table 1 represents the experimentally measured apparent axial modulus of the complete steel strand, obtained from the initial slope of its global tensile stress–strain response. Due to the helical configuration of the strand, its measured axial deformation includes both elastic elongation of the wires and geometric adjustment of the stranded assembly. Therefore, the measured apparent axial modulus was adopted in the three-stage constitutive relationship to reproduce the actual global tensile response of the steel strand.

The compression and tension stress–strain relationship for ECC employs the constitutive model, expressed as follows:

(2) $$y=\left\{\begin{array}{ll} 1.1x+0.5x^{5}-0.6x^{6} & \left(0\leq x\leq 1\right)\;\\ \;\frac{0.15x^{2}}{1-2x+1.15x^{2}} & \;\left(x\geq 1\right) \end{array}\right.$$

(3a) $$\sigma_{t}=\left\{\begin{array}{ll} E_{t}\varepsilon_{t} & \left(0\leq \varepsilon_{t}\leq \varepsilon_{\mathrm{km}}\right)\;\\ \left(c\frac{\varepsilon_{t}}{\varepsilon_{u}}+1-c\right)f_{\mathrm{tu}} & \left(\varepsilon_{\mathrm{km}}\leq \varepsilon_{t}\leq \varepsilon_{\mathrm{tu}}\right)\; \end{array}\right.$$

(3b) $$c=(f_{\mathrm{tu}}-f_{\mathrm{km}})\varepsilon_{\mathrm{tu}}/\left[(f_{\mathrm{tu}}-f_{\mathrm{km}}\right)f_{\mathrm{tu}}]$$

where *x* = *σ*/*f*_u_, *y* = *ε*/*ε*_u_; *f*_u_ is the peak compressive strength of ECC; ε_u_ is the strain corresponding to the peak compressive strength of ECC; *σ*_t_ and *ε*_t_ are the tensile stress and tensile strain of ECC, respectively; *f*_tu_ and *ε*_tu_ are the ultimate tensile strength and ultimate tensile strain of ECC, respectively; *f*_km_ and *ε*_km_ are the nominal cracking strength and cracking strain of ECC, respectively.

### 2.2. Establishment of the FE Model

**Figure 1:**
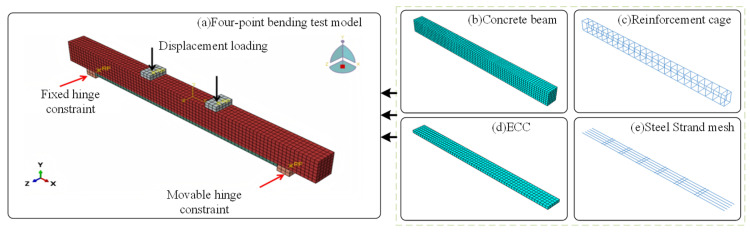
FE model of the reinforced beam.

The FE model is shown in Figure 1. A three-dimensional solid model was employed for numerical simulation of the flexural performance of RC beams strengthened with steel strand mesh-reinforced ECC. Concrete, ECC, and steel spacers were modeled by using C3D8R elements. Steel spacers were placed at the loading points of the beam to prevent stress concentration. Steel bars and steel strands were modeled by using the T3D2 element. All element sizes were set to 20 mm. The internal longitudinal reinforcing bars and stirrups were arranged within the RC beam according to the reinforcement layout of the reference specimens, while the steel strand mesh was embedded in the ECC strengthening layer at the bottom tensile face of the beam, as illustrated in Figure 1c,e.

In the section of contact settings, since no pull-out failure of the internal tensile reinforcement or pull-out failure of the steel strands from the ECC matrix was observed before flexural failure of the strengthened beams during the tests, the interfacial damage mainly occurred at the interface between the concrete substrate and the strengthening layer. Therefore, the bond between the concrete and the reinforcing bars, as well as that between the ECC matrix and the steel strands, was modeled using the embedded region method. The bearings and spacers were bonded to the surface of the RC beam through tie constraints.

The strengthened RC beam was anchored at the ends of the strengthening layer by using carbon fiber fabric wraps [24]. This effectively prevented end delamination during loading. In the FE model, the CFRP anchorage was not explicitly simulated by orthotropic CFRP elements. Instead, its anchoring effect was represented by tie constraints between the strengthening layer and the concrete substrate only within the CFRP-wrapped anchorage regions. The middle non-anchored region was modeled using cohesive surface interaction, allowing interfacial damage and slip to develop. The interface bond–slip behavior is defined by the bond–slip relationship model for steel strand mesh-reinforced ECC and concrete [25], which has been validated in previous experimental studies and is capable of capturing the nonlinear evolution, peak bond stress, and post-peak softening behavior of the interface. This simplification may restrict local debonding in the anchorage zones; however, the experimental beams did not exhibit premature end debonding, and the observed failure was dominated by flexural failure accompanied by concrete crushing or rupture of the strengthening layer. Therefore, the simplified anchorage treatment was considered acceptable for predicting the global flexural behavior of the strengthened beams.

(4) $$\tau =\left\{\begin{array}{ll} \tau_{u}{\left(\frac{s}{s_{u}}\right)}^{0.75} & s\leq s_{u}\\ \tau_{u}e^{-{\left(\alpha ((s/s_{u})-1)\right)}^{2}} & s>s_{u} \end{array}\right.$$

(5) $$\alpha_{b}=1/(\frac{G_{e}}{\tau_{u}s_{u}}-\frac{2}{3})$$

where *τ* is the bond stress corresponding to a slip displacement *s*; *τ*_u_ is the peak bond stress; *s*_u_ is the slip displacement corresponding to *τ*_u_; *α*_b_ is a coefficient determined based on experimental data; *G*_e_ is the interfacial strain energy.

### 2.3. Verification of the FE Model

To validate the FE model established in this paper, the numerical simulation for the previous test [17] on undamaged RC beams strengthened with steel strand mesh-reinforced ECC was conducted. A total of seven beam specimens were tested, including one unstrengthened RC beam and six strengthened RC beams. The strengthened beams were designed to investigate the effects of steel strand diameter, longitudinal steel strand reinforcement ratio, and ECC mix proportion. Based on the compression tests of three 150 mm × 150 mm × 150 mm concrete cubes, the measured average cubic compressive strength was 41.8 MPa. According to GB 50010–2010, the corresponding axial compressive strength was calculated as 27.7 MPa [26]. These measured concrete properties were adopted in the FE model. The yield strength of the longitudinal steel bar was 413.3 MPa, and that of the stirrups was 513.0 MPa.

The measured mechanical properties of the ECC mixtures and steel strands adopted in the FE validation are summarized in Table 1 and Table 2, respectively. The ECC used in the strengthening layer was a PVA fiber-reinforced engineered cementitious composite. According to the previous experimental study [26], the ECC matrix consisted of Portland cement, fly ash, silica fume, quartz sand, water, superplasticizer, thickener, and micro-polyvinyl alcohol (PVA) fibers.

Three ECC mixtures were adopted to investigate the influence of ECC material properties on the flexural strengthening effect. For each ECC mixture, the compressive strength (*f*_e_) and elastic modulus (*E*_e_) were obtained from compression tests, whereas the tensile cracking strength (*f*_km_), cracking strain (*ε*_km_), ultimate tensile strength (*f*_tu_), and ultimate tensile strain (*ε*_tu_) were determined from direct tensile tests on thin-plate specimens [26]. At least three identical specimens were tested for each mixture, and the values reported in Table 2 are expressed as mean ± standard deviation.

The geometry, reinforcement arrangement, strengthening configuration, boundary conditions, loading method, and material parameters of the FE model were kept consistent with those of the corresponding experimental beams. In the present paper, these test results were adopted only as benchmark data to verify the reliability of the proposed FE model before conducting the subsequent parametric analysis.

To avoid confusion between the simplified specimen labels used in the present FE validation and the specimen names reported in the previous experimental study [17], the correspondence between the two nomenclature systems is provided in Table 3.

**Figure 2:**
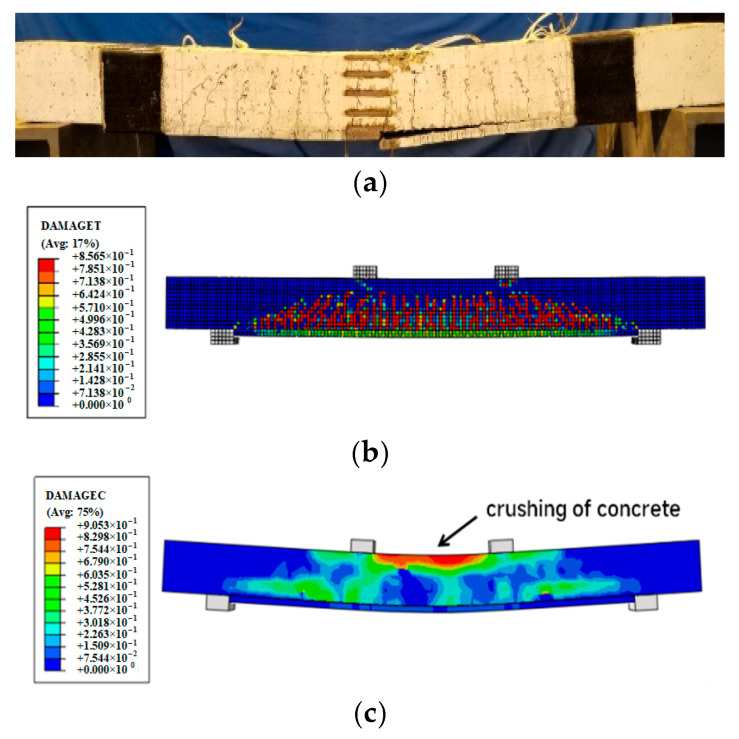

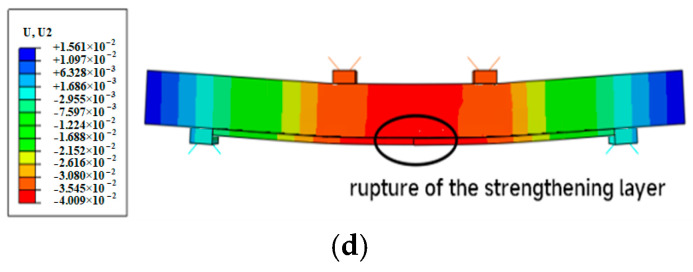
C-1 specimen failure mode comparison diagram: (**a**) Test failure mode of C-1 specimen, (**b**) C-1 specimen tensile damage distribution map, (**c**) C-1 specimen compressive damage distribution map, and (**d**) C-1 specimen vertical deformation distribution map.

**Figure 3:**
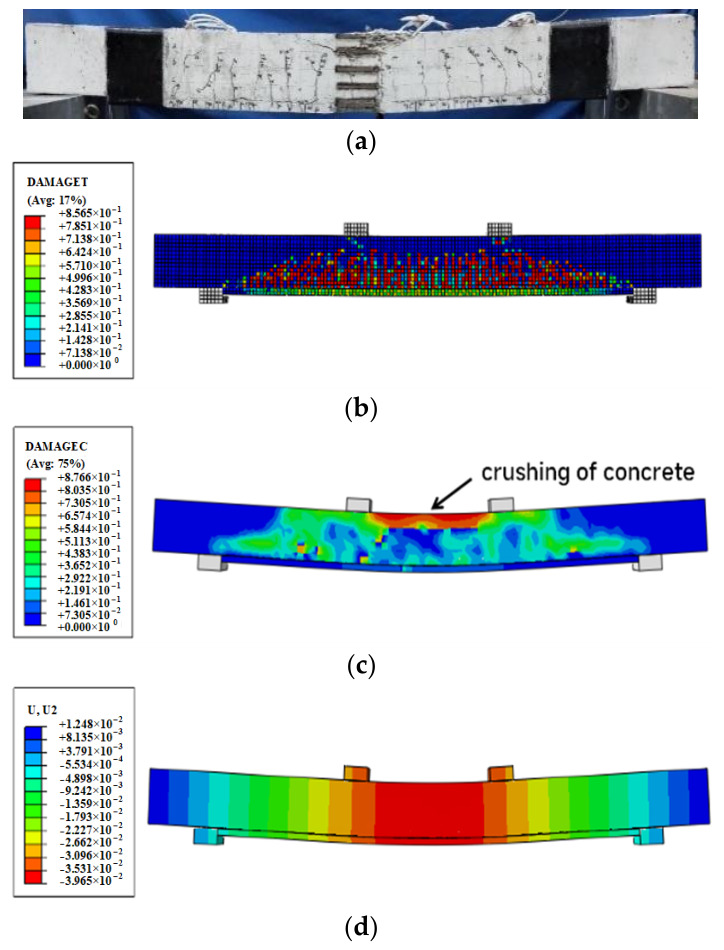
A-3 specimen failure mode comparison diagram: (**a**) Test failure mode of A-3 specimen, (**b**) A-3 specimen tensile damage distribution map, (**c**) A-3 specimen compressive damage distribution map, and (**d**) A-3 specimen vertical deformation distribution map.

Based on the FE results, the strengthened beams primarily exhibited two failure modes. In the first mode, the longitudinal steel bar in the RC beam yielded first. Subsequently, the concrete reached its ultimate compressive strain. After the reduction in load, concrete crushing occurred, and the steel strands at major cracks reached the ultimate tensile strain, leading to the rupture of the strengthening layer. In the second failure mode, the longitudinal steel bars also yielded first, followed by concrete crushing. However, the strengthening layer remained intact. Figure 2 and Figure 3 display the FE and test results that corresponded to the two failure modes, including the vertical deformation and compressive and tensile damage.

**Figure 4:**
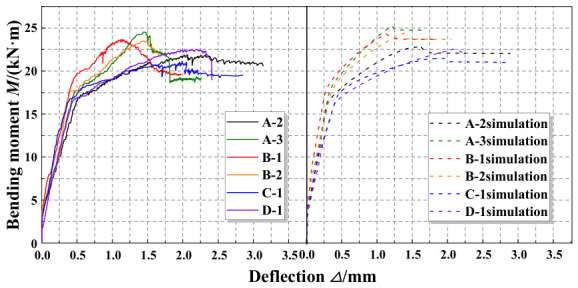
Comparison of the bending moment–midspan deflection curve of the strengthened RC beam specimens.

**Figure 5:**
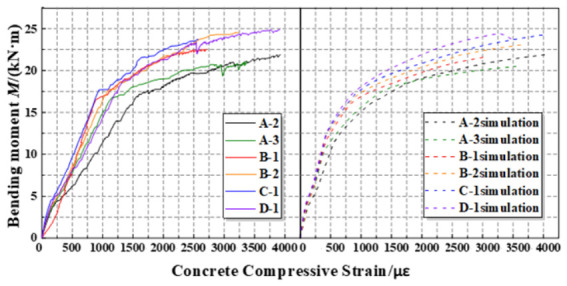
Comparison of the bending moment–midspan concrete compression strain curve of the strengthened RC beam specimens.

**Figure 6:**
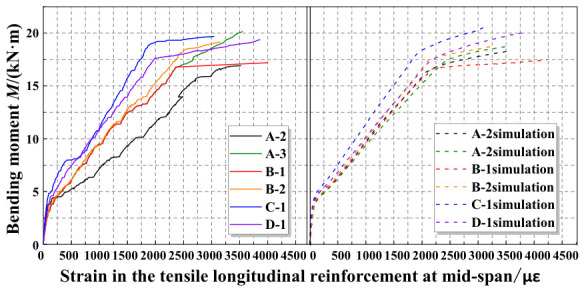
Comparison of the bending moment–strain curve of longitudinal reinforcement in the tension of strengthened RC beam specimens.

**Figure 7:**
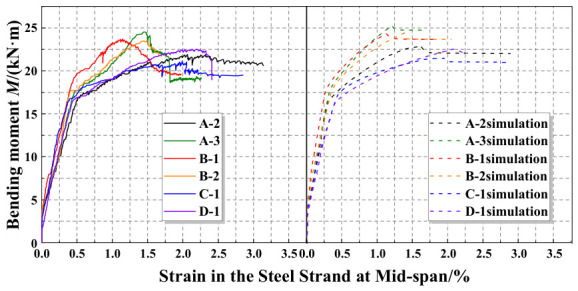
Comparison of the bending moment–strain curve of steel strand in the midspan of strengthened RC beam specimens.

The FE results of each specimen were compared with the experimental results, including the bending moment–deflection, bending moment–compressive strain of concrete, bending moment–strain of longitudinal steel bar, and bending moment–steel strand strain curves, as shown in Figure 4, Figure 5, Figure 6 and Figure 7. The results indicated that both sets generally match well. Specifically, for specimen A-2, cracks traversed the measurement zone of the strain gauges in the later stages of loading. This caused the strain of tensile steel bars measured in the experiment to develop faster than the simulated results.

In summary, the FE model provides a reasonable prediction of the flexural behavior of the strengthened RC beams, as indicated by the comparison of the failure modes, moment–deflection curves, strain responses, and quantitative validation indices listed in Table 4. Therefore, in the following sections, this model will be used for the parametric analysis of the flexural performance of beams.

## 3. Parametric Analysis

### 3.1. Parametric Research Design

A total of 38 simply supported beams were designed to further expand the range and types of influencing factors. Among these, nine were unstrengthened control RC beams, and 29 were non-destructive RC beams strengthened with steel strand mesh-reinforced ECC. The following seven material and geometric properties were key parameters. These affected the flexural performance of the beams in Table 5.

### 3.2. Influence of Material Parameters

#### 3.2.1. Influence of Cracking Strength of ECC

**Figure 8:**
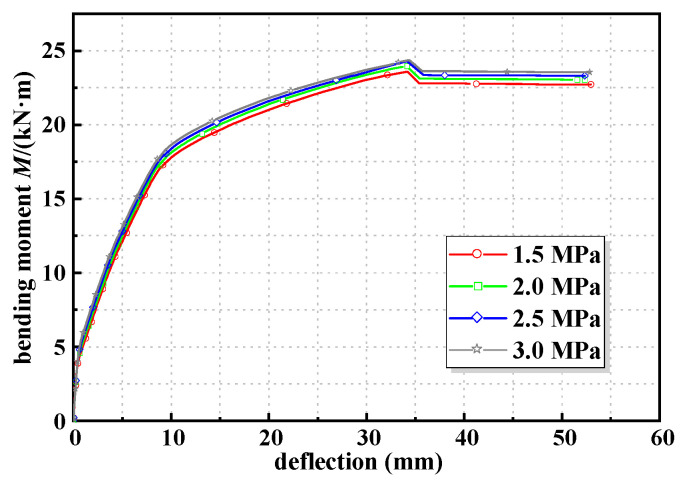
Moment–deflection curves with different ECC cracking strengths.

The simulation results of different cracking strengths are shown in Table 6. The moment–deflection curves for strengthened beams with different cracking strengths of ECC are shown in Figure 8. The results showed that an increasing cracking strength slightly enhanced the cracking, yield, and ultimate moment. In addition, the stiffness during the working stage with cracks of the strengthened beam was increased, while the stiffness at the elastic stage remained unchanged. Differently, the improvement in ductility of the strengthened beam decreased slightly with increasing the cracking strength of ECC. The force of the tension zone and the height of the compression zone in the cross-section were increased with increasing cracking strength. This decreased the deformation capacity of the beam and the improvement of ductility.

#### 3.2.2. Influence of Ultimate Tensile Strength of ECC

**Figure 9:**
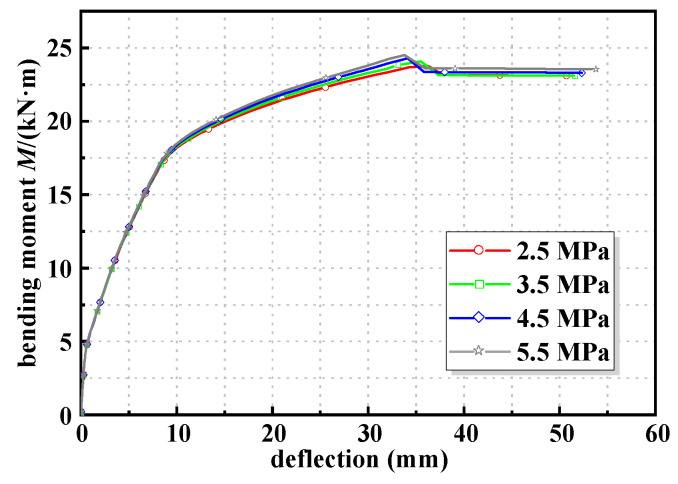
Moment–deflection curves with different ultimate tensile strengths of ECC.

The simulation results of different ultimate tensile strengths of ECC are shown in Table 7. The moment–deflection curves for strengthened beams with different ultimate tensile strengths of ECC are shown in Figure 9. As the ultimate tensile strength of ECC increased, the results showed the cracking moment of the beam remained unchanged, while the yield and ultimate moments increased continuously. This trend indicated that the stiffness after cracking was enhanced, but the stiffness before cracking remained unaffected. The ductility of the beams decreased slightly with the increase in ECC tensile strength. This phenomenon was caused by the enhanced deformation modulus of ECC after cracking. While maintaining its elastic modulus and ultimate tensile strain, this increase in deformation modulus boosted the stiffness of the strengthening layer after cracking. This resulted in a higher yield moment, ultimate moment, and stiffness of the beam after cracking. In addition, as the ultimate tensile strength of ECC increased, the force of the tension zone at ultimate conditions and the height of the compression zone also increased.

#### 3.2.3. Influence of Concrete Strength

**Figure 10:**
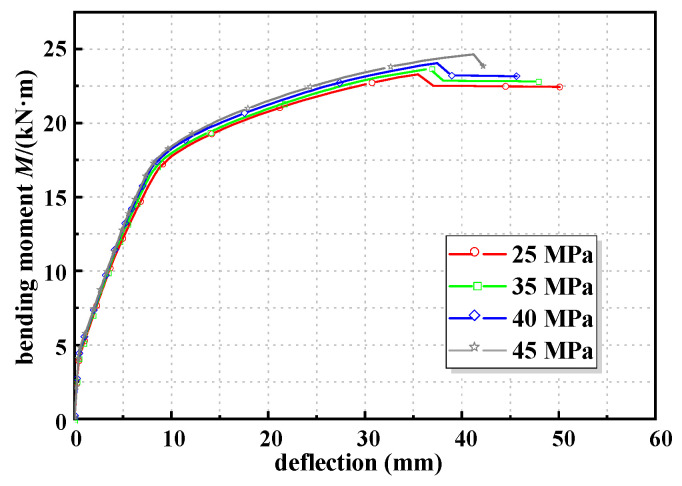
Moment–deflection curves of specimens with different concrete strengths.

The simulation results of specimens with different concrete strengths are shown in Table 8. The moment–midspan deflection curves for strengthened beams with different concrete strengths are shown in Figure 10. As the concrete strength increased, the enhancement in ductility increased. However, the rate of improvement in the stiffness, cracking, yield, and ultimate moment gradually diminished. In addition, the increase in concrete strength enhanced the deformation capacity, but the height of the compression zone and the yield displacement decreased. This allowed the strengthening layer to improve the ductility of the strengthened beam.

### 3.3. Influence of Geometric Parameters

#### 3.3.1. Effect of ECC Thickness

**Figure 11:**
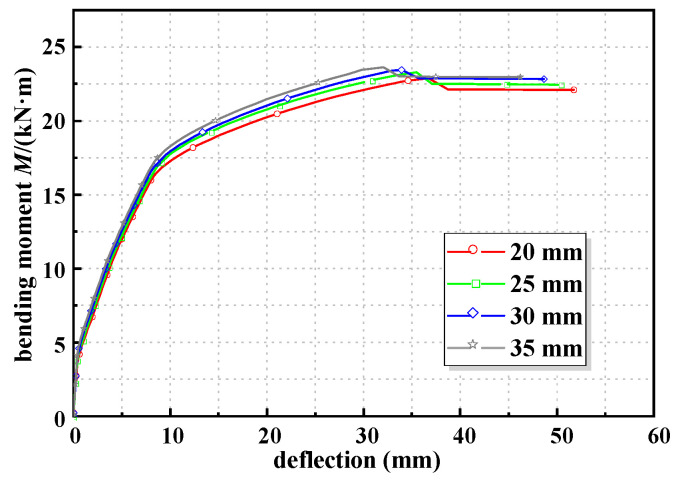
Moment–deflection curves of specimens with different ECC thicknesses.

The moment–midspan deflection curves for strengthened beams with different ECC thicknesses are shown in Figure 11. The simulation results of specimens with different ECC thicknesses are shown in Table 9. As the ECC thickness increased from 20 mm to 35 mm, the cracking moment, yielding moment, ultimate moment, and stiffness during the elastic and cracked working stages exhibited an approximately linear increase. However, the improvement in ductility gradually diminished. This was caused by the increase in the ECC thickness. It resulted in a larger section height and subsequently enhanced the total tensile force in the tension zone. Additionally, the ECC retained the load-carrying capacity after cracking and improved the overall mechanical properties of the beam. Meanwhile, the steel strand mesh-reinforced ECC demonstrated excellent crack-controlled capability. This enhanced the deformation capacity of the RC beam. However, with the increase in ECC thickness, the height of the compression zone also increased. This reduced the ultimate curvature and deformation capacity. As a result, the improvement in ductility diminished.

#### 3.3.2. Impact of Reinforcement Ratio of Longitudinal Steel Strand

**Figure 12:**
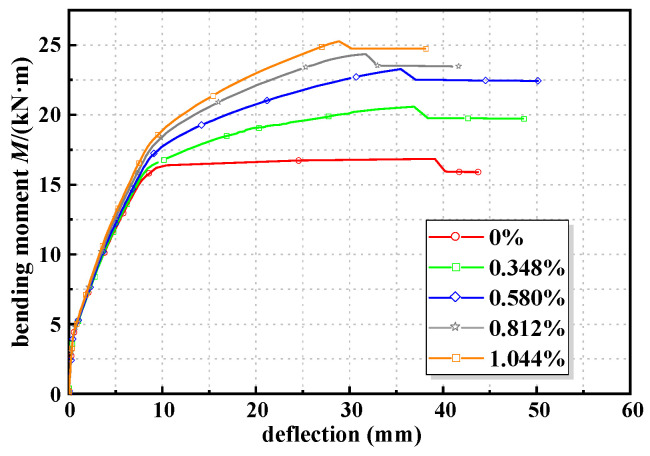
Moment–deflection curves of specimens with different reinforcement ratios of longitudinal steel strands.

Figure 12 presents the moment–deflection curves for specimens with varying reinforcement ratios of longitudinal steel strands. The simulation results are in Table 10. As the reinforcement ratio of longitudinal steel strands increased, the cracking moment, yield moment, ultimate moment, and flexural stiffness of the strengthened beams increased. This is because a higher steel strand ratio increases the tensile rigidity of the steel strand mesh-reinforced ECC layer. Under the same bending moment, a larger portion of the tensile force is carried by the strengthening layer, and the strain demand in the internal tensile steel bars is reduced. Consequently, the curvature and mid-span deflection of the strengthened beam decrease at the same load level, which explains the improvement in flexural stiffness. However, at the ultimate state, the increased tensile resultant provided by the steel strands shifts the neutral axis upward and increases the depth of the concrete compression zone. As a result, the ultimate curvature and deformation capacity decrease, leading to a reduction in the ductility coefficient. In addition, when the steel strand ratio becomes relatively high, the tensile strength of the steel strands cannot be fully utilized before concrete crushing, which explains the reduced growth rate of the ultimate moment.

#### 3.3.3. Impact of Reinforcement Ratio of Longitudinal Steel Bars

**Figure 13:**
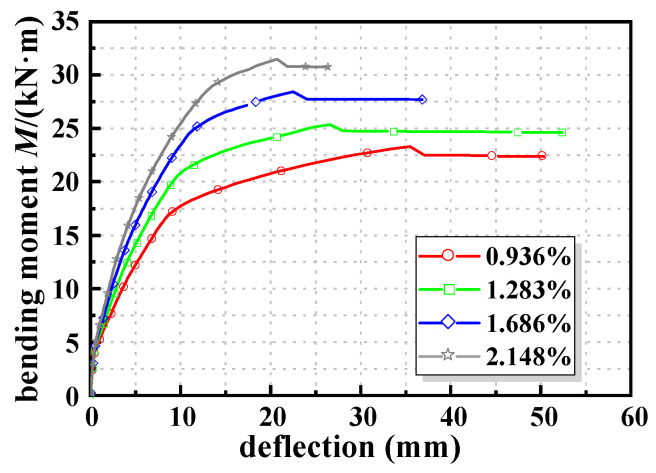
Moment–deflection curves of specimens with different reinforcement ratios.

Figure 13 shows the moment deflection curves for beams with different reinforcement ratios of longitudinal steel bars. Based on the FE results in Table 11, the strengthened beams exhibited an improvement in cracking moment, yield moment, ultimate moment, stiffness, and ductility. As the reinforcement ratio of steel bars increased, the cracking moment showed a significant improvement, while the improvement in other indicators decreased. After cracking the ECC, the increased reinforcement ratio of steel bars enhanced the restraint ability on beam cracks. This delayed the propagation of ECC cracks into the concrete. However, increasing the reinforcement ratio of steel bars raised the height of the compression zone of concrete. It resulted in a reduction in the deformation capacity of the beam and the utilization rate of the steel strands. Consequently, the improvement in yield moment, ultimate moment, and ductility was diminished.

#### 3.3.4. Impact of Section Height of the RC Beam

**Figure 14:**
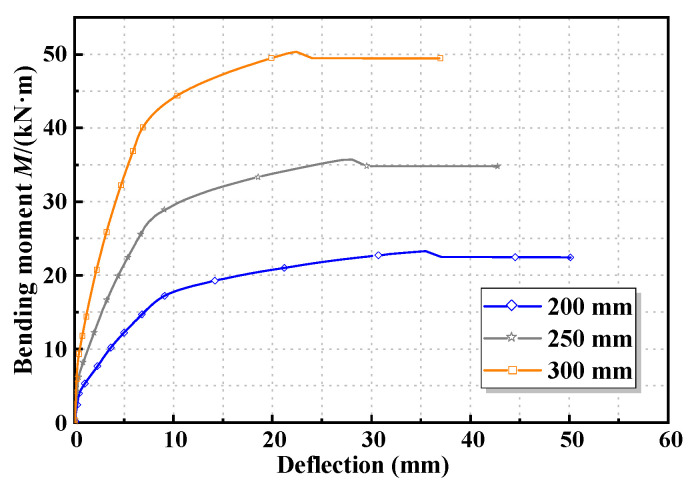
Moment–deflection curves of specimens with different heights of RC beam.

Figure 14 shows the bending moment deflection curves of strengthened beams with varying heights. Compared to the original RC beams, the strengthened beams enhanced the cracking moment, yield moment, ultimate moment, stiffness, and ductility in Table 12. As the height of the beam section increased, the magnitude of improvement for these parameters gradually diminished. When the reinforcement ratio of steel bars remained constant, the increase in the beam height enhanced the stiffness. In addition, it also increased the height of the compression zone in the strengthened beam section. This reduced its deformation capacity and decreased the strain in the strengthening layer during critical states. Consequently, the enhancement effects on cracking moment, yield moment, load-bearing capacity, stiffness, and ductility were diminished.

### 3.4. Parameter Sensitivity Analysis

To further identify the relative influence of different design parameters on the strengthening efficiency of strengthened RC beams, a normalized range-based sensitivity analysis was conducted. The performance index, including cracking moment *M*_cr_, yield moment *M*_s,y_, ultimate moment *M*_u_, elastic stage stiffness *B*_e_, cracked stage stiffness *B*_c_, and ductility coefficient *μ*_Δ_, was observed. It should be noted that min–max normalization itself is not a direct proof of parameter sensitivity. Instead, it was used in this study as a dimensionless preprocessing method to eliminate the influence of different units and numerical magnitudes among the design parameters and mechanical performance indicators. The parameter sensitivity was then evaluated according to the variation amplitude and slope tendency of the normalized response curves. The performance index and design parameter values are normalized using the following equation [27]:

(6) $$Z_{N}=\frac{x_{i}-\min(x)}{\max(x)-\min(x)}$$

where *Z*_N_ is the normalized value of the performance index or design parameter, satisfying 0 ≤ *Z*_N_ ≤ 1; x*_i_* is a specific actual value of the performance index or design parameter; and min(*x*) and max(*x*) are the minimum and maximum actual values of the performance index or design parameter, respectively.

**Figure 15:**
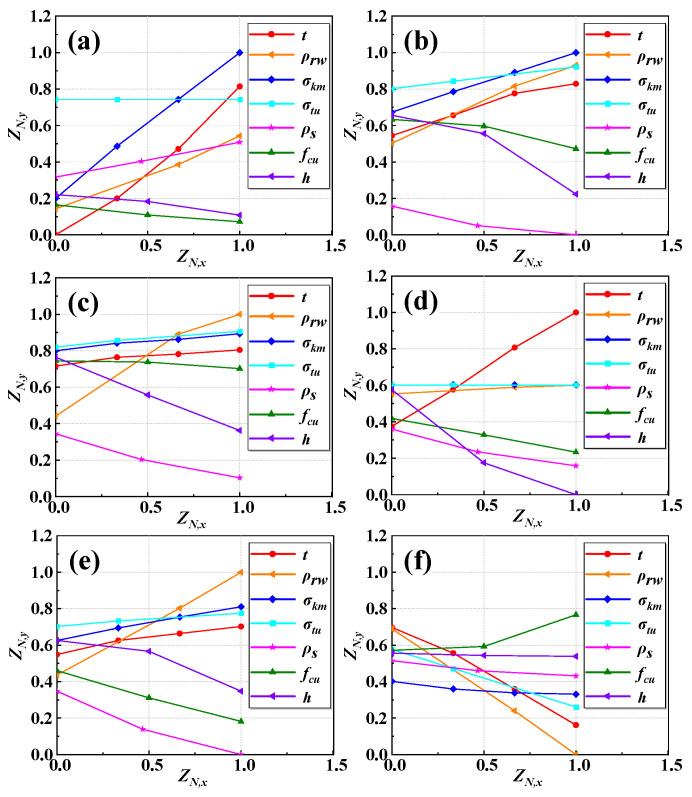
Normalized results of evaluation indicators for different design parameters of strengthened RC beams: (**a**) Evaluation metric *M*_cr_, (**b**) evaluation metric *M*_s,y_, (**c**) evaluation metric *M*_u_, (**d**) evaluation metric *B*_e_, (**e**) evaluation metric *B*_c_, (**f**) evaluation metric *μ*_△_.

The normalized result of the performance index and design parameter is shown in Figure 15. The vertical axis *Z*_N,y_ and horizontal axis *Z*_N,x_ represent the normalized values of the evaluation index and design parameters, respectively.

Through comprehensive analysis, it can be concluded that the improvement effect of *Mc,r* on the beam was most sensitive to the strengthening layer and the cracking strength of the ECC. This is because increasing the strengthening layer significantly improved the resistance moment of the beam section, and a higher cracking strength meant the material could withstand larger tensile strength without cracking. Increasing the longitudinal steel strands was equivalent to increasing the tensile reinforcement; therefore, the reinforcement ratio of longitudinal steel strands was the next most influential factor.

The enhancement effect of *M*_s,y_ was most sensitive to the height of the beam. Without changing the reinforcement ratio, increasing the height of the beam increased the height of the compression zone. Consequently, the deformation and strain of steel bars under yield load were significantly reduced in the strengthened beam. This diminished the contribution of the strengthening layer.

The enhancement effect on *M*_u_ was most sensitive to the reinforcement ratio of longitudinal strands, followed by the beam height and the reinforcement ratio of steel bars. This occurred because the strength utilization of longitudinal strands was high at the ultimate load. Increasing the quantity of strands markedly increased the total tensile force in the tension zone. This significantly boosted the ultimate moment.

The enhancement effect at the elastic stage *B*_e_ was most sensitive to the thickness of the strengthening layer and the height of the beam, followed by the reinforcement ratio of steel bars and concrete strength. The mechanical properties of ECC had almost no effect on its enhancement. Increasing the thickness of the strengthening layer significantly increased the resistance moment of the beam section. Increasing the height of the beam also markedly improved the sectional stiffness of the beam. This significantly decreased the enhancement of sectional stiffness.

The enhancement effect on *B*_c_ was most sensitive to the reinforcement ratio of the longitudinal steel strand, followed by the sensitivity to characteristic parameters of the beam. Increasing the reinforcement ratio of longitudinal steel strands significantly enhanced the crack-controlled capacity of the strengthening layer. This markedly improved Bc. Conversely, increasing the reinforcement ratio of steel bars, concrete strength, or height of the RC beam all enhanced the stiffness of the beam but diminished the effect of the strengthening layer on improving the stiffness of the original beam.

The enhancement effect on *μ*_△_ was most sensitive to the thickness of the strengthening layer and the reinforcement ratio of the longitudinal steel strand, followed by the sensitivity to the ultimate tensile strength of the ECC. All strengthened beams experienced crushing failure. As the ECC thickness and the reinforcement ratio of steel strands increased, the height of the compressed zone under ultimate conditions significantly increased. This led to poorer deformation capacity, resulting in a marked reduction in the improvement of ductility. Increasing the ultimate tensile strength of ECC also increased the height of the compressed zone under ultimate conditions.

## 4. The Bending Performance Prediction of Strengthened Beams

The calculation procedure in this section is based on sectional analysis. First, the strain distribution of the strengthened section is determined according to the plane-section assumption. Then, the stresses of concrete, tensile steel bars, steel strands, and ECC are obtained from the corresponding constitutive relationships. The internal force equilibrium of the section is used to determine the height of the compression zone. Finally, the flexural capacity and stiffness are calculated from moment equilibrium and curvature–deflection relationships, respectively.

### 4.1. Basic Assumptions and Material Constitutive Relationships

(1) The positive section strain of RC beams strengthened with the steel strand mesh-reinforced ECC that satisfied the plane section assumption [24].
(2) After the cracking of the concrete in the tension zone, the tensile steel bars and the steel strand mesh-reinforced ECC jointly bore the tensile force.
(3) Prior to the strengthened beam reaching its ultimate flexural capacity state, no relative slip occurred between concrete and steel bars or between steel strands and the ECC. Moreover, considering the effective coordination between the original beam and the strengthening layer during testing, no premature debonding was observed. Therefore, it is concluded that deformation coordination existed [24] between the original beam and the strengthening layer, with no relative slip.
(4) The material constitutive relationships are described in Section 2.1.

### 4.2. Bearing Capacity Prediction

#### 4.2.1. Calculation Formulas

**Figure 16:**
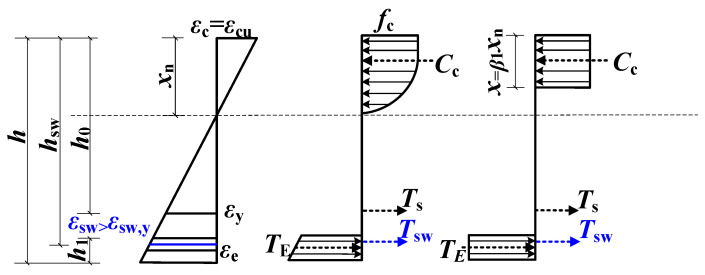
Distribution of cross-section strain and strength of strengthened RC beams. Note: *h* is the height of the strengthened beam section; *h*_sw_ is the distance from the steel strand to the top of the compression zone; *h*_0_ is the effective height of the concrete section; *h*_1_ the thickness of the strengthening layer; *ε*_c_ is the concrete compressive strain; *ε*_cu_ is the ultimate compressive strain value of concrete under non-uniform compression; *x*_n_ is the height of the compression zone; *ε*_sw_ is the tensile strain in the steel strand; *ε*_sw,y_ is the strain corresponding to the nominal yield strength of the steel strand; *ε*_y_ is the tensile strain of the steel bar at yield stress; *ε*_e_ is tensile strain of the strengthening layer; *T*_s_ is tensile force provided by the reinforcement; *T*_sw_ the tensile force provided by the steel strand; *T*_E_ is the tensile force provided by the strengthening layer; *C*_c_ is the compressive force provided by the concrete.

Under reasonable strengthening, the failure mode of the specimen was as follows. At the ultimate state of the beam, the concrete reached its ultimate compressive strain after the yielding of the tensile steel bars and steel strands. At this point, *ε*_c_ = *ε*_cu_, *ε*_sw_ > *ε*_sw,y_. The strain and stress distribution of the cross-section in the strengthened RC beam is shown in Figure 16.

Li et al. [17] performed a calculation method for the ultimate bearing capacity of the RC beam strengthened with a steel strand mesh-reinforced ECC. However, this calculation method still presented the following issues. Firstly, it employed a three-branch constitutive model for steel strands. When calculating the strand strength, it required the determination of whether the strand strain was in the second or third stage during beam failure. Additionally, it also considered the strain lag of the strengthening layer. This increased computational complexity. The strain of the ECC reached the strain-hardening stage at the ultimate state of the beam. The influence of strain lag for the ECC can be disregarded. Consequently, this paper simplifies these aspects as follows:

(1) The influence of the ECC strain lag is ignored.
(2) Simplify the three-stage constitutive model by proposing a two-stage constitutive model for tensile steel strands, expressed as follows:

(7) $$\left\{\begin{array}{ll} E_{\mathrm{sw}1}\varepsilon_{\mathrm{sw}} & 0\;<\varepsilon_{\mathrm{sw}}\leq \varepsilon_{\mathrm{sw},y}\\ f_{\mathrm{sw},y}+E_{\mathrm{sw}2}\;(\varepsilon_{\mathrm{sw}}-\varepsilon_{\mathrm{sw},y}) & \varepsilon_{\mathrm{sw},y}<\varepsilon_{\mathrm{sw}}\leq \varepsilon_{\mathrm{sw},u} \end{array}\right.$$

where *f*_sw,y_ is the nominal yield strength of the steel strand, approximately 85% of the ultimate tensile strength; *ε*_sw,y_ is the strain corresponding to the nominal yield strength, approximately 40% of the ultimate tensile strain; *E*_sw1_ and *E*_sw2_ are the secant moduli of the strand for the two stages, respectively.

Subsequently, the simplified calculation equation for the ultimate flexural capacity of the strengthened beam is derived as follows:

(8) $$\begin{array}{l} \alpha_{1}f_{cd}bx=f_{yd}A_{s}+A_{\mathrm{sw}}\left[f_{\mathrm{sw},y}+E_{\mathrm{sw}2}\left(\varepsilon_{\mathrm{cu}}\frac{\beta_{1}h_{\mathrm{sw}}-x}{x}-\varepsilon_{\mathrm{sw},y}\right)\right]\\ \;+A_{E}\left(c\varepsilon_{\mathrm{cu}}\frac{2\beta_{1}-\beta_{1}h_{1}-2x}{2x\varepsilon_{e,u}}+1-c\right)\sigma_{e,u} \end{array}$$

(9) $$\begin{array}{l} M_{u}=f_{yd}A_{s}\left(h_{0}-\frac{x}{2}\right)+A_{\mathrm{sw}}\left[f_{\mathrm{sw},y}+E_{\mathrm{sw}2}\left(\varepsilon_{\mathrm{cu}}\frac{\beta_{1}h_{\mathrm{sw}}-x}{x}-\varepsilon_{\mathrm{sw},y}\right)\right]\left(h_{\mathrm{sw}}-\frac{x}{2}\right)\\ \;+A_{E}\sigma_{e,u}\left(c\varepsilon_{\mathrm{cu}}\frac{2\beta_{1}h-\beta_{1}h_{1}-2x}{2x\varepsilon_{e,u}}+1-c\right)\left(h-\frac{x}{2}-\frac{h_{1}}{2}\right) \end{array}$$

where *f*_cd_ is the design value of the axial compressive strength for concrete; *ε*_0_ is the concrete compressive strain corresponding to compressive strength *f*_cd_; *ε*_cu_ is the ultimate compressive strain of concrete under non-uniform compression; *f*_u_ is the peak strength; *A*_s_ is the total cross-sectional area of the longitudinal steel bars; *f*_yd_ is the design value of tensile strength for steel bar; and *c* can be calculated by Equation (3b).

#### 4.2.2. Verification of the Ultimate Bending Capacity Formula

**Figure 17:**
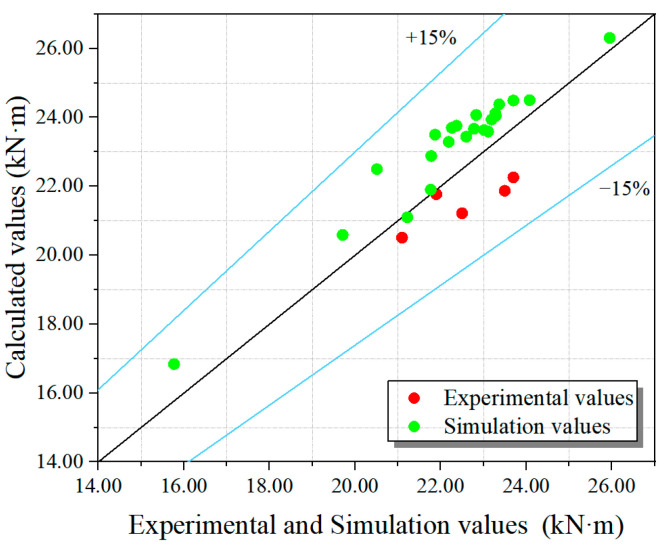
The degree of dispersion between the calculated values of the ultimate bending capacity formula of the RC beam and the experimental and simulation values.

The results of the FE simulation (*M*_su_) and experimental values (*M*_tu_) were compared with the calculated values (*M*_cu_) from the proposed formula in Figure 17. These figures indicate that the simplified formula achieves high accuracy in calculating the ultimate load-bearing capacity of strengthened beams.

### 4.3. Calculation of the Quantity of Boundary Steel Strand for Bending Reinforcement

#### 4.3.1. Calculation of Minimum Steel Strand Quantity for Bending Reinforcement

The minimum steel strand usage for RC beams strengthened with steel strand mesh-reinforced ECC must satisfy the following conditions:

(1) To prevent under-reinforced failure, the reinforcement ratio *ρ* must ensure that the steel strands do not yield before cracking of the strengthened beam:

(10) $$\rho ={A_{s}}^{\prime }/bh_{0}\geq \rho_{\min}$$

where *ρ*_min_ is the minimum reinforcement ratio, determined according to the Chinese Code [23]; *A*_s_′ is the cross-sectional area of the equivalent steel bar obtained based on the principle of stiffness equivalence, expressed as follows:

(11) $${A_{s}}^{\prime }=A_{s}+\frac{E_{\mathrm{sw}}}{E_{s}}A_{\mathrm{sw}}$$

Solving Equations (10) and (11) simultaneously yields the minimum steel strand strengthening area, *A*_sw_. One minute is required to ensure the steel strands do not yield before the cracking of beams:

(12) $$A_{\mathrm{sw},\min1}=\frac{E_{s}}{E_{\mathrm{sw}}}(\rho_{\min}bh_{0}-A_{s})$$

(2) To ensure that strands remain intact before the concrete of the beam is crushed, the minimum strengthening area *A*_sw,min2_ is derived as follows:

From the mechanical equilibrium relationship:

(13) $$\alpha_{1}f_{\mathrm{cd}}bx=f_{\mathrm{yd}}A_{s}+f_{\mathrm{sw},u}A_{\mathrm{sw},\min2}+\sigma_{e}A_{e}$$

(14) $$A_{\mathrm{sw},\min2}=\frac{\alpha_{1}f_{\mathrm{cd}}bx-f_{y}A_{s}-\sigma_{e}A_{e}}{f_{\mathrm{sw},u}}$$

Under the assumption of a plane section, the following equation can be derived:

(15) $$\frac{\varepsilon_{\mathrm{cu}}}{x_{n}}\;=\frac{\varepsilon_{\mathrm{sw},u}}{h_{\mathrm{sw}}-x_{n}}\;=\frac{\varepsilon_{e}}{h-\frac{h_{1}}{2}-x_{n}}\;$$

From the above equation, the following equation can be derived:

(16) $$x=\frac{\beta_{1}h_{\mathrm{sw}}\varepsilon_{\mathrm{cu}}}{\varepsilon_{\mathrm{cu}}+\varepsilon_{\mathrm{sw},u}}$$

(17) $$\varepsilon_{e}=\varepsilon_{\mathrm{cu}}\frac{2\beta_{1}h-\beta_{1}h_{1}-2x}{2x}$$

Therefore, the tensile stress provided by the ECC is as follows:

(18) $$\sigma_{e}=c\varepsilon_{\mathrm{cu}}(\frac{2\beta_{1}h-\beta_{1}h_{1}-2x}{2x\varepsilon_{u}}+1-c)\;\sigma_{e,u}$$

Substituting Equations (16) and (18) into Equation (14) yields *A*_sw,min2_. Where *α*_1_ is the equivalent rectangular stress block coefficient of concrete, and *β*_1_ is the coefficient for the equivalent compressive zone depth of concrete. According to the Chinese Code [23], *α*_1_ = 1.0 and *β*_1_ = 0.8 for the concrete strength level considered in this study.

To validate the accuracy of the formula for calculating the required quantity of steel strands for flexural strengthening at boundary conditions, additional specimens were added to simulate different reinforcement ratios of the steel strands. Except for the usage of the steel strand, all other parameters were identical to the original specimens. When each specimen failed due to concrete crushing, the strength and strain results for the strengthening bars and steel strands were shown in Table 13.

As shown in Table 11, when the number of steel strands was 1, the steel strands broke during concrete crushing. However, when the number of steel strands exceeded 1, the strengthening bars and steel strands reached their yield strength, and the steel strand was not broken. Substituting the parameters of the selected steel bars and strands into the formula yields *A*_sw,min2_ = 5.32 mm^2^ under this condition.

#### 4.3.2. Calculation of Maximum Steel Strand Quantity for Bending Reinforcement

To prevent over-reinforced failure in beams under bending, the maximum usage of steel strands must be analyzed.

From the mechanical equilibrium condition comes the following equation:

(19) $$\alpha_{1}f_{\mathrm{cd}}bx=f_{\mathrm{yd}}A_{s}+f_{\mathrm{sw},y}A_{\mathrm{sw},\max}+\sigma_{e}A_{e}$$

(20) $$A_{\mathrm{sw},\max}=\frac{\alpha_{1}f_{\mathrm{cd}}bx-f_{\mathrm{yd}}A_{s}-\sigma_{e}A_{e}}{f_{\mathrm{sw},y}}$$

Under the assumption of a plane section, we obtain the following:

(21) $$\frac{\varepsilon_{\mathrm{cu}}}{x_{n}}=\frac{\varepsilon_{\mathrm{sw},y}}{h_{\mathrm{sw}}-x_{n}}=\frac{\varepsilon_{e}}{h-h_{1}/2-x_{n}}$$

From the above equation, we obtain the following:

(22) $$x=\frac{\beta_{1}h_{\mathrm{sw}}\varepsilon_{\mathrm{cu}}}{\varepsilon_{\mathrm{cu}}+\varepsilon_{\mathrm{sw},y}}$$

(23) $$\varepsilon_{e}=\varepsilon_{\mathrm{cu}}\;\frac{2\beta_{1}h-\beta_{1}h_{1}-2x}{2x}$$

Therefore, the tensile stress provided by the ECC is as follows:

(24) $$\sigma_{e}=c\varepsilon_{\mathrm{cu}}(\frac{2\beta_{1}h-\beta_{1}h_{1}-2x}{2x\varepsilon_{u}}+1-c)\sigma_{e,u}$$

Substituting Equations (22) and (24) into Equation (20) yields A_sw,max_.

It should be noted that Equations (11)–(18) and (19)–(24) are established to define the lower and upper bounds of steel strand reinforcement, corresponding to the ultimate strength state and yield strength state, respectively. Although the formulations are similar in form, they are based on different strength criteria and parameter definitions.

As shown in Table 13, when the cross-sectional area of the steel strand was less than 26.10 mm^2^, both the steel bars and steel strands reached their yield strength. When the cross-sectional area of the steel strand exceeded 26.10 mm^2^, the steel bars yielded, but the steel strands failed to reach the yield strength. Substituting the parameters of the selected steel bars and strands into the formula yields *A*_sw,max_ = 27.64 mm^2^ under this condition.

### 4.4. Calculation and Analysis of Bending Stiffness

#### 4.4.1. Formula for Calculating Bending Stiffness of Cross-Sections of Strengthened RC Beam

The short-term bending stiffness Bs of the strengthened RC beam was calculated using Equation (28), while Equations (25)–(27) and (29)–(32) were used to determine the equivalent reinforcement area, effective section height, strain non-uniformity coefficient, equivalent reinforcement ratio, and equivalent tensile stress required in Equation (28).

**Figure 18:**
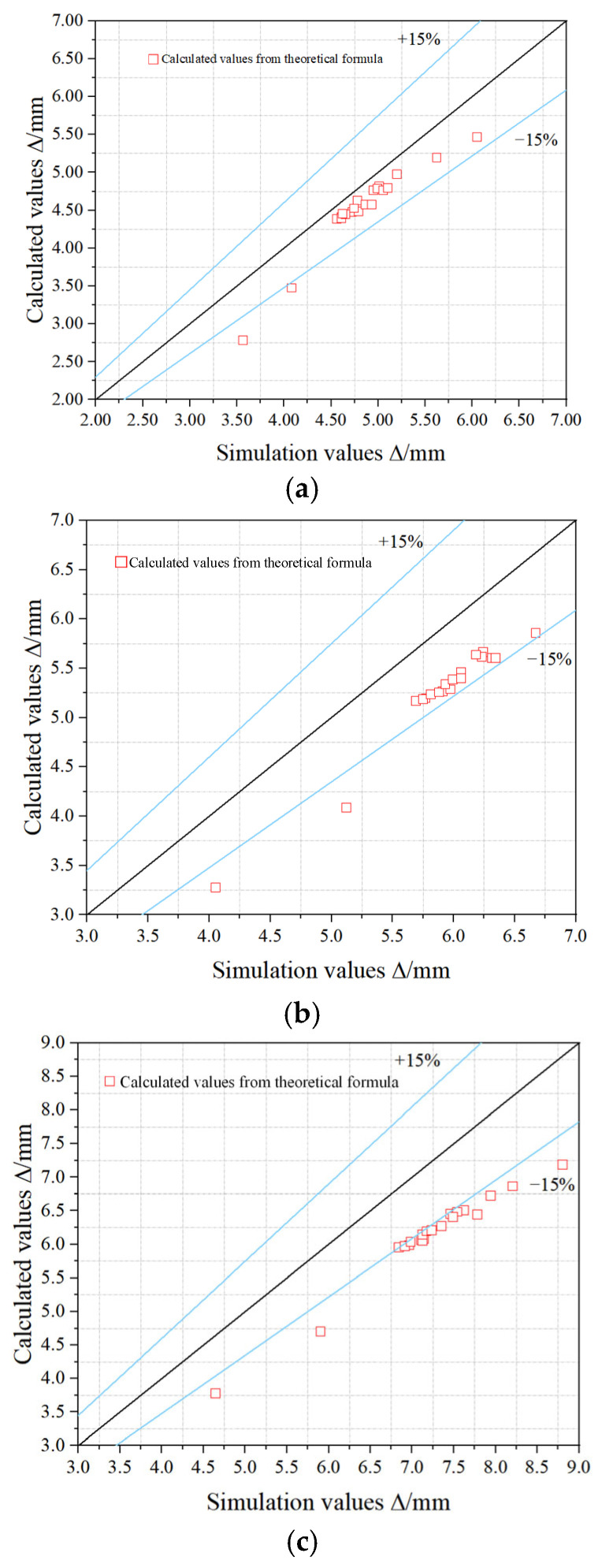
The dispersion between the simulation value (∆_sm_) of the deflection and the calculated value (∆_cm_) of the formula in the different yielding moments (*M*_s,y_): (**a**) 70% *M*_s,y_, (**b**) 80% *M*_s,y_, (**c**) 90% *M*_s,y_.

Li et al. [17] proposed a method for calculating the flexural stiffness (*B*_s_) of the RC beams strengthened with steel strand mesh-reinforced ECC. The dispersion between the simulation value (∆_sm_) of the deflection and the calculated value (∆_cm_) of the formula in the different yielding moments (*M*_s,y_) is shown in Figure 18. The general calculation method for deflection (∆) is shown in Equation (25). In the equation, M and l are the moment and beam span, respectively. When specimens were at 80% *M*_s,y_ and 90% *M*_s,y_ states, the simulation deflection values differ significantly from the formula-calculated values. This discrepancy is mainly attributed to the multiple-cracking and strain-hardening characteristics of the ECC. The post-cracking tangent modulus of the ECC is difficult to determine accurately, and directly adopting the theoretical tangent modulus may lead to obvious deviations in the calculated deflection, especially when the strengthened beam approaches the yield stage. Therefore, a strain-bond adjustment coefficient, *η*_ε_, was introduced in this study. The effect of the ECC on the flexural stiffness was incorporated into the coefficient ψ, so that the influence of the ECC strengthening layer on the stiffness of the specimens could be reasonably considered and the stiffness calculation formula could be corrected. Based on the results of the specimens with different ECC cracking strengths, *η*_ε_ = 0.92 was adopted for calculations.

(25) $$\Delta =\frac{23Ml^{2}}{216B_{s}}$$

This led to the simplified bending stiffness calculation formula as follows:

(26) $${A_{s}}^{\prime }=A_{s}+\frac{A_{sw}E_{sw}}{E_{s}}$$

(27) $$E_{s}A_{s}x_{1}=E_{sw}A_{sw}\left(h_{sw}-h_{0}-x_{1}\right)$$

(28) $${h_{0}}^{\prime }=h_{0}+x_{1}$$

(29) $$B_{s}=\frac{E_{s}{A_{s}}^{\prime }{{h_{0}}^{\prime }}^{2}}{1.15\psi +0.2+6\alpha_{E}\rho }$$

(30) $$\psi =\eta_{\varepsilon }(1.1-\frac{0.65f_{tk}}{\rho_{te}\sigma_{ss}})$$

(31) $$\rho =\frac{{A_{s}}^{\prime }}{b{h_{0}}^{\prime }}$$

(32) $$\rho_{te}=\frac{{A_{s}}^{\prime }}{0.5bh}$$

(33) $$f_{ss}=\frac{M}{0.87{A_{s}}^{\prime }{h_{0}}^{\prime }}$$

where *E*_s_ is the elastic modulus of the steel bars; *h*_0_’ is the effective height of beam calculation section; *A*_s_′ is the total equivalent cross-sectional area of tensile steel bars; *η*_ε_ is the strain-bond adjustment factor; *B*_s_ is the short-term stiffness of RC flexural member; *ψ* is the strain non-uniformity coefficient of longitudinal tensile steel bars between cracks; *α*_E_ is the ratio of the elastic modulus of steel to that of concrete; *ρ*_te_ is the equivalent longitudinal tensile reinforcement ratio of the strengthened beam calculated based on the effective tensile concrete cross-sectional area; and *f*_ss_ is the equivalent longitudinal tensile strength of the reinforced beam.

#### 4.4.2. Verification of Bending Stiffness Calculation Formula

**Figure 19:**
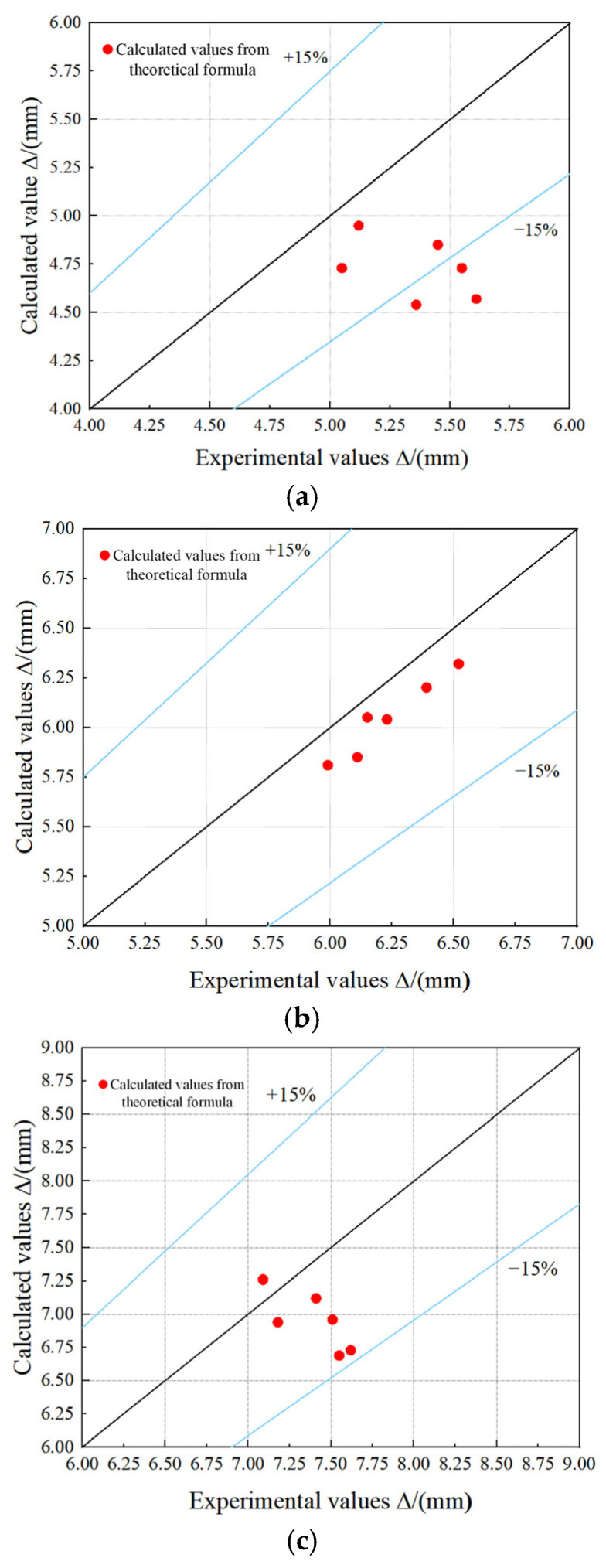
The degree of dispersion between the calculated values of the stiffness formula of the RC beam and the experimental values: (**a**) 70% *M*_s,y_, (**b**) 80% *M*_s,y_, (**c**) 90% *M*_s,y_.

As shown in Figure 19, a scatter plot was plotted to illustrate the dispersion between the calculated deflection values of the test specimen (∆_ct_) and the experimental values of the test specimen (∆_st_) in the different yielding moments (*M*_s,y_). The results indicated good agreement between the formula-calculated values and test results under all conditions.

**Figure 20:**
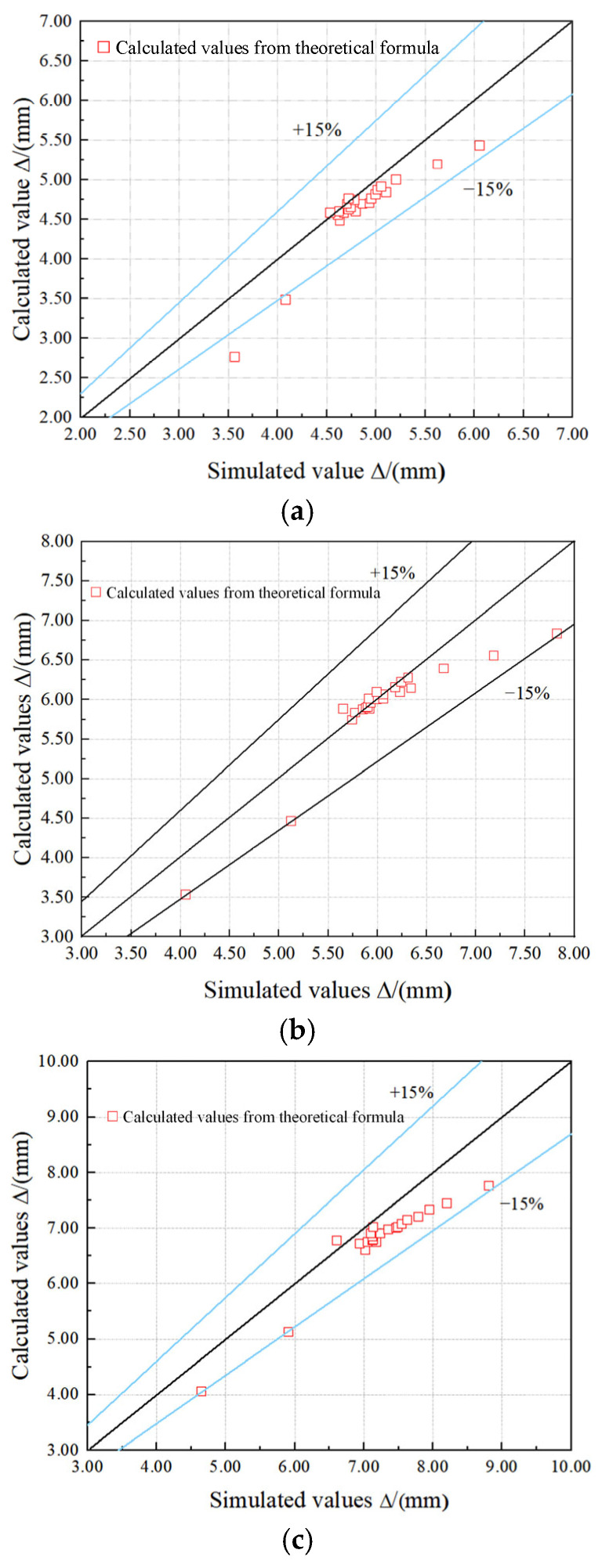
The degree of dispersion between the calculated values of the stiffness formula of the RC beam and the simulation values: (**a**) 70% *M*_y_, (**b**) 80% *M*_y_, (**c**) 90% *M*_y_.

Figure 20 illustrates the dispersion between the calculated deflection values of the simulation specimen (∆_cm_) and the simulation values of the simulation specimen (∆_sm_) in the different yielding moments (*M*_s,y_). The results indicated that the simplified stiffness calculation formula offers superior accuracy.

## 5. Conclusions

Through numerical simulation and theoretical analysis, the flexural performance of undamaged RC beams strengthened with strand mesh-reinforced ECC was investigated. The influence parameters included the ECC thickness, reinforcement ratio of longitudinal steel strands, cracking strength of ECC, ultimate tensile strength of ECC, longitudinal reinforcement ratio of the RC beam, concrete strength, and height of the RC beam. The main conclusions were as follows:

(1) The FE model established for undamaged RC beams strengthened with strand mesh-reinforced ECC showed good agreement with experimental results. Results indicated that this reinforcement method significantly enhanced the cracking moment, yield load, ultimate load-carrying capacity, stiffness, and ductility of undamaged RC beams.
(2) The influence of various parameters on the cracking, yield, and ultimate moment of strengthened beams was as follows. An increase in ECC thickness and cracking strength significantly enhanced the improvement of the cracking moment. An increase in the reinforcement ratio of longitudinal steel strands markedly boosted the improvement of the yield and ultimate moment. An increase in the longitudinal reinforcement ratio of the RC beam slightly increased the improvement of the cracking moment but reduced that of the yield and ultimate moment. An increase in concrete strength and the height of the RC beam diminished the improvement of the cracking, yield, and ultimate moment.
(3) Influence patterns of various factors on the stiffness and ductility of strengthened beams. Increasing the ECC thickness and reinforcement ratio of the longitudinal steel strand significantly boosted the stiffness increase of the beam. In contrast, increasing the longitudinal reinforcement ratio of the RC beam, concrete strength, and height of the RC beam decreased the increase in stiffness. The ultimate tensile strength of the ECC slightly raised the increase in stiffness during the cracked working stage. Regarding ductility, increasing concrete strength enhanced the ductility improvement of the strengthened beam, while increasing the other six parameters reduced the ductility improvement.
(4) The proposed formulas included the calculation of the boundary steel strand quantity, a simplified formula of ultimate bending capacity, and a formula for the bending stiffness of cross-sections of strengthened RC beams. These formulas showed good agreement with experimental and numerical simulation results, validating the accuracy of the proposed formulas.

## Figures and Tables

**Table 1:** Tensile properties of the high-strength steel strands [17].

*d* (mm)	*A*_sw_ (mm^2^)	*E*_rw_ (GPa)	*f*_swu_ (MPa)	*ε*_swu_ (%)
3.0	4.35	139	1919.02	2.96
3.6	6.43	109	1521.21	3.47
4.5	9.65	130	1706.46	3.37

Note: *A*_sw_ is the cross-sectional area of the steel strand, and *E*_rw_ is the elastic modulus of the steel strand; *f*_swu_ is the ultimate tensile strength of the steel strand; *ε*_swu_ is the ultimate tensile strain of the steel strand.

**Table 2:** Measured mechanical properties of the ECC mixtures used for FE validation (mean ± standard deviation).

ECC Formula	*f*_e_ (MPa)	*E*_e_ (GPa)	*f*_km_ (MPa)	*ε*_km_ (%)	*f*_tu_ (MPa)	*ε*_tu_ (%)
1	37.3 ± 2.2	14.1 ± 0.4	1.37 ± 0.07	0.025 ± 0.003	2.18 ± 0.12	1.88 ± 0.25
2	46.5 ± 2.8	14.6 ± 0.4	1.91 ± 0.10	0.035 ± 0.004	2.81 ± 0.15	0.75 ± 0.10
3	36.6 ± 2.2	14.3 ± 0.4	1.86 ± 0.09	0.032 ± 0.004	2.30 ± 0.12	2.47 ± 0.30

Note: *f*_e_ is the compressive strength of ECC, and *E*_e_ is the elastic modulus of ECC, respectively; *f*_km_ and *ε*_km_ are the tensile cracking strength and cracking strain, respectively; *f*_tu_ and *ε*_tu_ are the ultimate tensile strength and ultimate tensile strain, respectively.

**Table 3:** Correspondence between specimen labels used in this study and those in the previous experimental study.

Specimen Label in This Study	Specimen Name in Previous Experimental Study	Main Feature
A-2	SF1-0.580–3.0	ECC formula F1; *d_sw_* = 3.0 mm; *ρ_sw_* = 0.580%
A-3	SF1-0.812–3.0	ECC formula F1; *d_sw_* = 3.0 mm; *ρ_sw_* = 0.812%
B-1	SF2-0.580–3.0	ECC formula F2; *d_sw_* = 3.0 mm; *ρ_sw_* = 0.580%
B-2	SF3-0.580–3.0	ECC formula F3; *d_sw_* = 3.0 mm; *ρ_sw_* = 0.580%
C-1	SF1-0.535–4.5	ECC formula F1; *d_sw_* = 4.5 mm; *ρ_sw_* = 0.515%
D-1	SF1-0.686–3.6	ECC formula F1; *d_sw_* = 3.6 mm; *ρ_sw_* = 0.686%

Note: *d_sw_* is the nominal diameter of the longitudinal steel strand; *ρ_sw_* is the reinforcement ratio of the longitudinal steel strands.

**Table 4:** Comparison of characteristic loads obtained from tests and simulations.

Specimen ID	*M_cr_* (kN·m)		McrexpMcrsim	*M_y_* (kN·m)		MyexpMysim	*M_u_* (kN·m)		MuexpMusim
	$M_{cr}^{exp}$	$M_{cr}^{sim}$		$M_{y}^{exp}$	$M_{y}^{sim}$		$M_{u}^{exp}$	$M_{u}^{sim}$	
L0	2.8	2.7	1.04	13.1	12.7	1.03	15.6	15.0	1.04
A-2	3.9	3.8	1.03	16.7	16.5	1.01	23.3	21.9	1.06
A-3	4.0	4.6	0.87	17.3	17.5	0.99	24.4	24.5	1.00
B-1	4.1	4.4	0.93	17.5	17.7	0.99	24.3	23.7	1.03
B-2	3.9	4.1	0.95	16.8	17.4	0.97	24.4	23.5	1.04
C-1	4.0	3.8	1.05	15.9	16.7	0.95	21.5	21.1	1.02
D-1	4.3	2.5	1.72	16.0	16.7	0.96	22.4	22.5	1.00
Average error									1.03

Note: *M*_cr_ is the cracking moment; *M*_y_ is the yield moment; *M*_u_ is the ultimate moment; *M*^exp^ is the experimental value; *M*^sim^ is the simulated value.

**Table 5:** Range of parameter analysis.

Parameters	Values	Basic Values
Cracking strength of ECC *f*_km_ (MPa)	2, 2.5, and 3	1.5
Ultimate tensile strength of ECC *f*_tu_ (MPa)	3.5, 4.5, and 5.5	2.5
Concrete strength *f*_cu_ (MPa)	35, 40, and 45	25
ECC thickness (mm)	20, 25, 30, and 35	25
Reinforcement ratio of the longitudinal steel strand *ρ*_rw_ (%)	0.348, 0.812, and 1.044	0.58
Reinforcement ratio of the longitudinal steel bars *ρ*_s_ (%)	1.283, 1.686, and 2.148	0.936
Section height of the RC beam (mm)	250, and 300	200

**Table 6:** Simulation results of different ECC cracking strength specimens.

*f*_km_ (MPa)	*M*_cr_ (kN·m)	*M*_s,y_ (kN·m)	*M*_u_ (kN·m)	*μ*_△_
1.5	3.86	16.78	23.59	3.97
2.0	4.06	17.18	23.94	3.91
2.5	4.24	17.56	24.12	3.88
3.0	4.42	17.95	24.38	3.87

Note: *M*_cr_ is the cracking moment; *M*_s,y_ is the yield moment; *M*_u_ is the ultimate moment; *μ*_△_ is the ductility coefficient, which can be calculated by the ratio of the deflection corresponding to *M*_u_ to the deflection corresponding to *M*_s,y_.

**Table 7:** Simulation results with different ultimate tensile strengths of ECC.

*f*_tu_ (MPa)	*M*_cr_ (kN·m)	*M*_s,y_ (kN·m)	*M*_u_ (kN·m)	*μ*_△_
2.5	4.24	17.24	23.76	4.22
3.5	4.24	17.39	24.07	4.07
4.5	4.24	17.56	24.12	3.88
5.5	4.24	17.67	24.49	3.77

**Table 8:** Simulation results of specimens with different concrete strengths.

*f*_cu_ (MPa)	*M*_cr_ (kN·m)	*M*_s,y_ (kN·m)	*M*_u_ (kN·m)	*μ*_△_
25	3.86	16.72	23.29	4.19
35	3.99	17.03	23.67	4.37
40	4.13	17.26	24.04	4.52
45	4.24	17.38	24.41	4.65

**Table 9:** Simulation results of specimens with different ECC thickness.

Thickness (mm)	*M*_cr_ (kN·m)	*M*_s,y_ (kN·m)	*M*_u_ (kN·m)	*μ*_△_
20	3.72	16.32	22.88	4.39
25	3.86	16.72	23.29	4.19
30	4.05	17.15	23.44	3.91
35	4.29	17.34	23.63	3.63

**Table 10:** Simulation results of specimens with different longitudinal reinforcement ratios of steel strands.

*ρ*_rw_ (%)	*M*_cr_ (kN·m)	*M*_s,y_ (kN·m)	*M*_u_ (kN·m)	*μ*_△_
0	3.77	15.54	16.84	4.82
0.348	3.82	16.1	20.5	4.38
0.580	3.86	16.72	23.29	4.19
0.812	3.99	17.2	24.3	3.74
1.044	4.1	17.7	25.2	3.4

**Table 11:** Simulation results for specimens with different longitudinal reinforcement ratios.

*ρ*_s_ (%)	*M*_cr_ (kN·m)	*M*_s,y_ (kN·m)	*M*_u_ (kN·m)	*μ*_△_
0.936	3.86	16.72	23.29	4.19
1.283	4	20.31	25.37	2.87
1.686	4.12	24.46	28.44	1.95
2.148	4.27	28.31	31.49	1.42

**Table 12:** Simulation results for specimens with different heights of RC beam.

Height (mm)	*M*_cr_ (kN·m)	*M*_s,y_ (kN·m)	*M*_u_ (kN·m)	*μ*_△_
200	3.86	16.72	23.29	4.19
250	5.67	27.25	35.71	3.86
300	7.8	40.08	50.36	3.27

**Table 13:** The strength and strain results for the strengthening bars and steel strands.

Sectional Area of the Steel Strand/mm^2^	Relative Values of Strength and Strain During Concrete Crushing			
	*σ*_s_/*f*_y_	*ε*_s_/*ε*_y_	*σ*_sw_/*f*_sw,y_	*ε*_sw_/*ε*_sw,y_
0	—	—	—	—
4.35 (1)	1	11.98	Tensile rupture	Tensile rupture
6.70 (2)	1	10.53	1.16	2.05
13.05 (3)	1	9.98	1.14	1.77
17.40 (4)	1	9.46	1.13	1.42
21.75 (5)	1	9.04	1.09	1.33
26.10 (6)	1	8.65	1.04	1.14
30.45 (7)	1	7.23	0.99	0.99
34.80 (8)	1	6.32	0.98	0.95
39.15 (9)	1	3.99	0.96	0.87

## Data Availability

The original contributions presented in this study are included in the article. Further inquiries can be directed to the corresponding authors.

## Nomenclature

*σ*_sw_	The tensile stress of steel strands
*ε*_sw_	The tensile strain of steel strands
*E*_sw*i*_	The elastic modulus of the steel strands at three stages, *i* = 1, 2, 3 (MPa)
ε_sw1_	The strain values of 40% of the ultimate tensile strain of steel strands
ε_sw2_	The strain values of 60% of the ultimate tensile strain of steel strands
ε_sw3_	The strain values of 100% of the ultimate tensile strain of steel strands
*σ*_sw*i*_	The corresponding stress values corresponding to ε_sw*i*_, *i* = 1, 2, 3 (MPa)
*f*_u_	The peak compressive strength of ECC (MPa)
ε_u_	The strain corresponding to the peak compressive strength of ECC
*ε*_t_	The tensile strain of ECC
*f*_tu_	The ultimate tensile strength of ECC (MPa)
*ε*_tu_	The ultimate tensile strain of ECC
*f*_km_	The nominal cracking strength of ECC (MPa)
*ε*_km_	The cracking strain of ECC
*s*	The slip of the steel strand (mm)
*τ*	The bond stress corresponding to *s* (MPa)
*τ*_u_	The peak bond stress (MPa)
*s*_u_	The slip displacement corresponding to *τ*_u_ (mm)
*α*_b_	The coefficient determined based on experimental data
*G*_e_	The interfacial strain energy
*f*_e_	The compressive strength of ECC (MPa)
*E*_e_	The elastic modulus of ECC (GPa)
*A*_sw_	The cross-sectional area of the steel strands (mm^2^)
*E*_rw_	The elastic modulus of the steel strand (GPa)
*f*_swu_	The ultimate tensile strength of the steel strand (MPa)
*ε*_swu_	The ultimate tensile strain of the steel strand
*f*_cu_	The axial compressive strength of concrete (MPa)
*ρ*_rw_	The reinforcement ratio of the longitudinal steel strand (%)
*ρ*_s_	The reinforcement ratio of the longitudinal steel bars (%)
*M*_cr_	The cracking moment (kN·m)
*M*_s,y_	The yield moment (kN·m)
*M*_u_	The ultimate moment (kN·m)
*μ*_△_	The ductility coefficient
*Z*_N_	The normalized value of the performance index or design parameter
x*_i_*	The specific actual value of the performance index or design parameter
min(*x*)	The minimum actual values of the performance index or design parameter
max(*x*)	The maximum actual values of the performance index or design parameter
*h*	The height of the strengthened beam section (mm)
*h*_sw_	The distance from the steel strand to the top of the compression zone (mm)
*h*_0_	The effective height of the concrete section; *h*_1_ is the thickness of the strengthening layer (mm)
*ε*_c_	The concrete compressive strain
*ε*_cu_	The ultimate compressive strain value of concrete under non-uniform compression
*x*_n_	The height of the compression zone (mm)
*ε*_sw,y_	The strain corresponding to the nominal yield strength of the steel strand
*ε*_y_	The tensile strain of the steel bar at yield stress
*ε*_e_	The tensile strain of the strengthening layer
*T*s	The tensile force provided by the reinforcement (kN)
*T*_sw_	The tensile force provided by the steel strands (kN)
*T*_E_	The tensile force provided by the strengthening layer (mm)
*C*_c_	The compressive force provided by the concrete (kN)
*f*_sw,y_	The nominal yield strength of the strand is approximately 85% of the ultimate tensile strength
*f*_cd_	The design value of axial compressive strength for concrete (MPa)
*ε*_0_	The concrete compressive strain corresponding to *f*_c_
*A*_s_	The total cross-sectional area of the longitudinal steel bars (mm^2^)
*f*_yd_	The design value of tensile strength for the steel bar (MPa)
*M*_su_	The FE simulation value of ultimate bending capacity (kN·m)
*M*_tu_	The experimental value of ultimate bending capacity (kN·m)
*M*_cu_	The calculated value of ultimate bending capacity (kN·m)
*ρ*_min_	The minimum reinforcement ratio (%)
*A*_s_′	The cross-sectional area of the equivalent steel bar obtained based on the principle of stiffness equivalence (mm^2^)
*∆*_sm_	The simulation value of the deflection (mm)
*∆*_cm_	The calculated value of the formula for the different yielding moments (mm)
*h*_0_’	The effective height of the beam calculation section (mm)
*A*_s_′	The total equivalent cross-sectional area of tensile steel bars (mm^2^)
*η*_ε_	The strain-bond adjustment factor
*B*_s_	The short-term stiffness of the RC flexural member (N/mm)
*ψ*	The strain non-uniformity coefficient of longitudinal tensile steel bars between cracks
*α*_E_	The ratio of the elastic modulus of steel to that of concrete
*E*_s_	The elastic modulus of the steel bars (GPa)
*ρ*_te_	The equivalent longitudinal tensile reinforcement ratio of the strengthened beam, calculated based on the effective tensile concrete cross-sectional area (%)
*f*_ss_	The equivalent longitudinal tensile strength of the reinforced beam (MPa)
